# Intraosseous Mucoepidermoid Carcinoma of the Anterior Mandible: A Case Report

**DOI:** 10.7759/cureus.25036

**Published:** 2022-05-16

**Authors:** Himsikhar Khataniar, Siddharth Senthil, Sumit S Deep, Rakesh Ramesh, Inchara Y K

**Affiliations:** 1 Surgical Oncology, St. Johns Medical College and Hospital, Bengaluru, IND; 2 Pathology, St. Johns Medical College and Hospital, Bengaluru, IND

**Keywords:** mandibulectomy, mucoepidermoid carcinoma, misdiagnosis, mandibular tumor, ameloblastoma

## Abstract

Intraosseous mucoepidermoid carcinoma (MEC) is a rare neoplasm, generally presenting in the posterior mandible and occurring in the 3rd-5th decade. This condition may mimic ameloblastoma both clinically and radiologically, with challenges in diagnosis. We present the case of a 51-year-old female who presented with a history of swelling over her jaw for one month. On examination, the mass involved the outer table of the mandible, from the right canine to the left first premolar. The swelling was hard, non-tender, with a nodular surface. A PET CT scan showed a multiloculated cystic lesion in the anterior mandible. An orthopantomogram (OPG) depicted a lytic lesion in the anterior mandible with outer table involvement and was suspected to be ameloblastoma. The patient underwent segmental mandibulectomy, neo-mandible reconstruction surgery with an osteomyocutaneous free fibular flap (from the right leg), and split skin grafting over the donor site. The patient recovered well. However, contrary to our suspected diagnosis, the final histopathological report showed features suggestive of mucoepidermoid carcinoma. Hence, mucoepidermoid carcinoma can be misdiagnosed as ameloblastoma due to similar clinico-radiological features. Histopathology is confirmatory and needs to be reviewed to confirm the diagnosis.

## Introduction

Mucoepidermoid carcinoma (MEC) is the most common type of salivary gland malignancy seen in adults. MEC is described by Stewart et al. as having an epidermal component and a mucus-secreting component [1]. Eighty-six percent of such cases are seen in the parotid gland, followed by submandibular (8%), sublingual (0.4%), and minor palatine salivary glands [2].

Intraosseous MECs are rare neoplasms, mostly detected in the posterior part of the mandible, and seen in the age group of 30-50 with slight female predilection [3]. The origin of this tumor could be from ectopic salivary gland tissue, submucosal glands with intraosseous extension, or transformed mucous cells from odontogenic cysts and maxillary sinus [3]. It presents usually as a painless mass with a gradually increasing volume and is perceived by the patients themselves within a year or less of onset [4].

This tumor is of clinical significance as it may metastasize to the gnathic bones, resulting in a poor prognosis. This tumor is often misdiagnosed clinically and radiographically as a benign odontogenic tumor or cyst. Thus, it is essential to perform histopathology of the resected sample and look for signs of malignancy to confirm the diagnosis of MEC. The most common treatment modality is radical surgical resection, which shows better cure rate results than curettage or enucleation due to the high probability of metastasis [3].

## Case presentation

**Figure 1:**
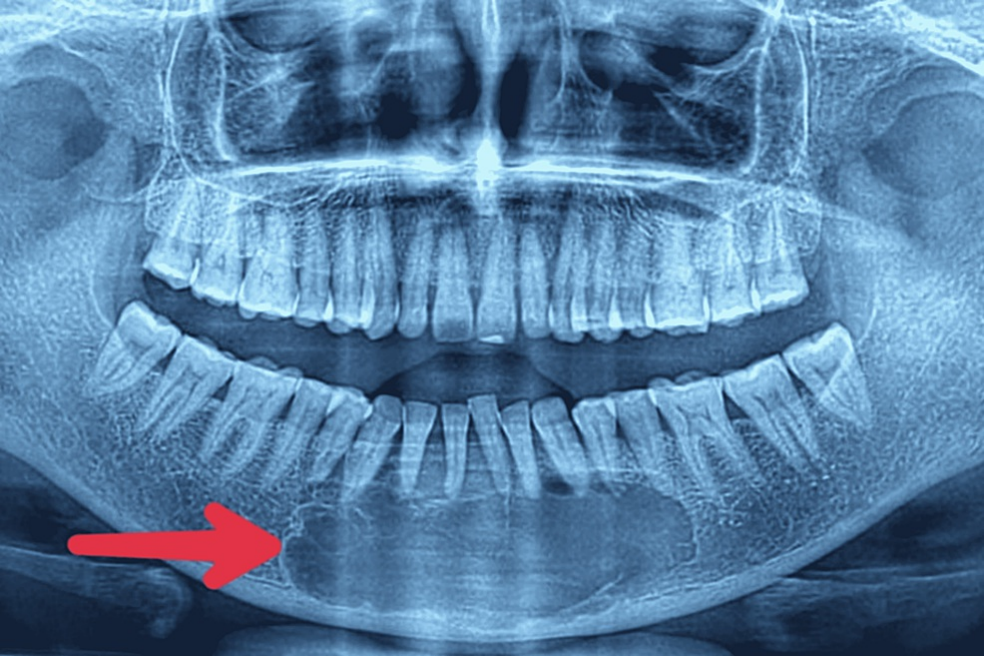
OPG X-ray showing multiloculated cystic lesion (red arrow) in the anterior mandible measuring 4.7 cm × 2.5 cm × 3.1 cm with no cortical breach

A 51-year-old female presented to the outpatient department of a hospital with complaints of swelling over her jaw for one month. The swelling was insidious in onset, rapidly progressive but not associated with any pain, trismus, dysphagia, difficulty in chewing, cough, hemoptysis, loosening of teeth, weight loss, or loss of appetite. The patient is a known case of carcinoma of the breast and had undergone right breast modified radical mastectomy (MRM) in 2015 along with adjuvant chemotherapy and hormonal therapy for five years. On examination, swelling of around 6 cm × 3 cm was present in the mandible, involving the outer angle, extending from the right canine to the left first premolar. The swelling was hard, non-tender, with a nodular surface. There was no ulceration or discharge, no absent teeth, and no cervical lymphadenopathy. The PET CT scan suggested a 4.7 cm × 2.5 cm × 3.1 cm multiloculated cystic lesion in the anterior mandible, involving the roots of bilateral incisors, canine and left premolars. An orthopantomogram (OPG) was done in which a lytic lesion was noted in the anterior mandible with involvement of the outer angle, suspected to be ameloblastoma (Figure 1).

**Figure 2:**
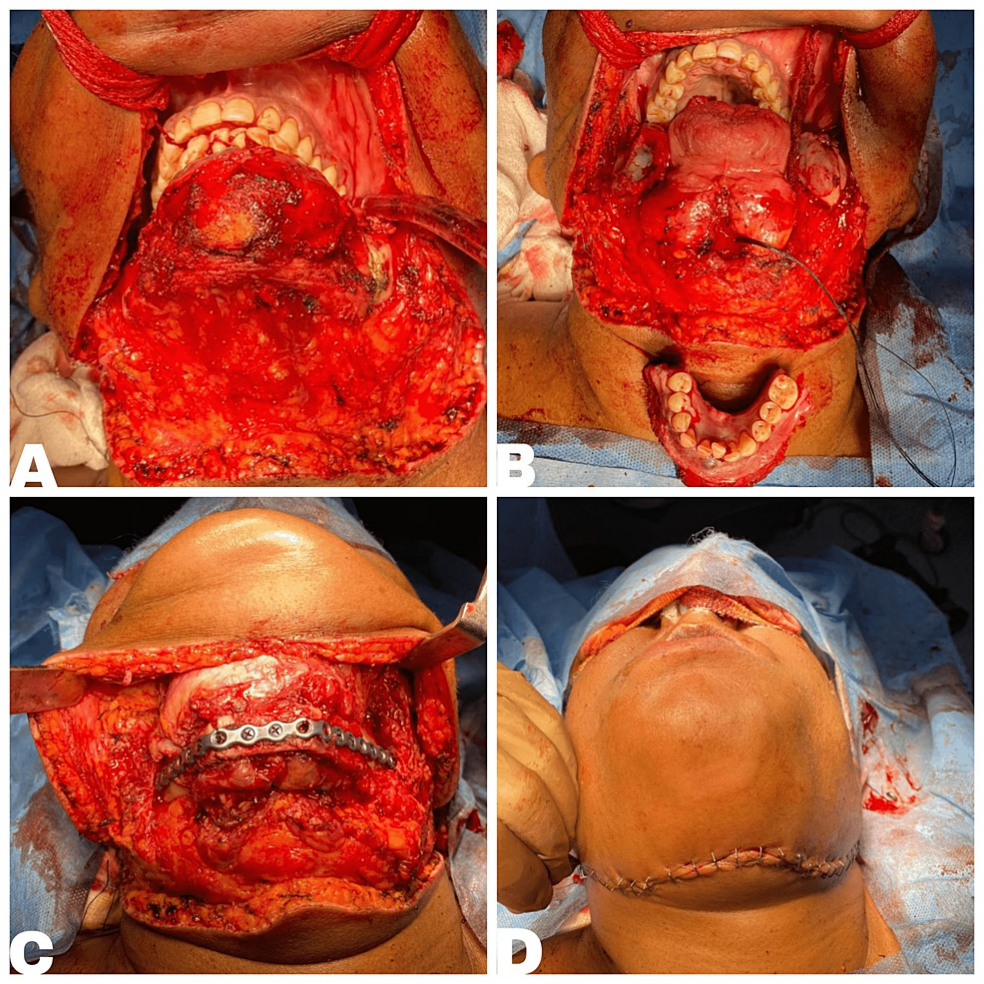
Images depicting the steps of surgery in tumor resection (A) Tumor exploration, (B) segmental mandibulectomy, (C) surgical plating, and (D) surgical site closure

After performing necessary preoperative evaluations, the patient with the help of multi-departmental cooperation, underwent segmental mandibulectomy, neo-mandible reconstruction with osteomyocutaneous free fibular flap (from the right leg), and split skin grafting over the donor site under general anesthesia (Figure 2).

**Figure 3:**
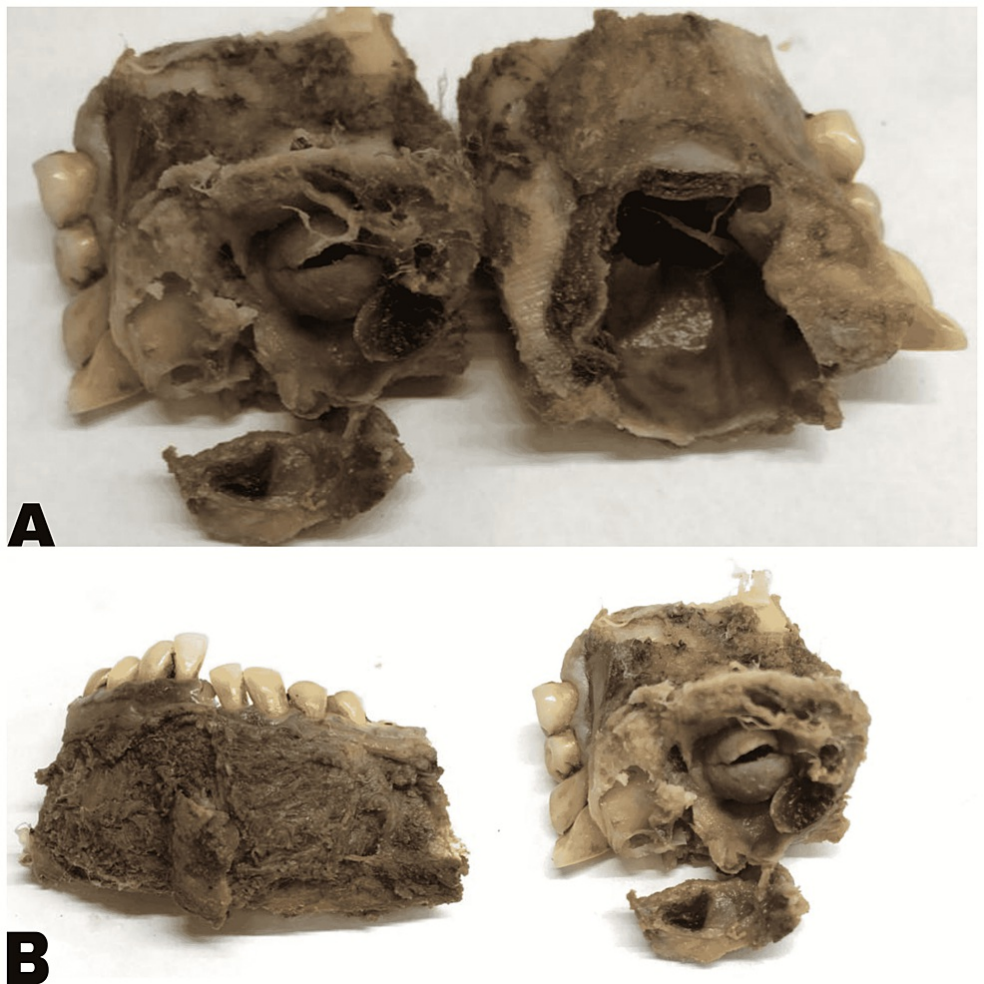
Images (A) and (B) depicting gross morphology of the resected tumor showing intraosseous cystic lesion. Note the septations within the cyst.

The resected mandible was sent for histopathological examination (Figure 3).

**Figure 4:**
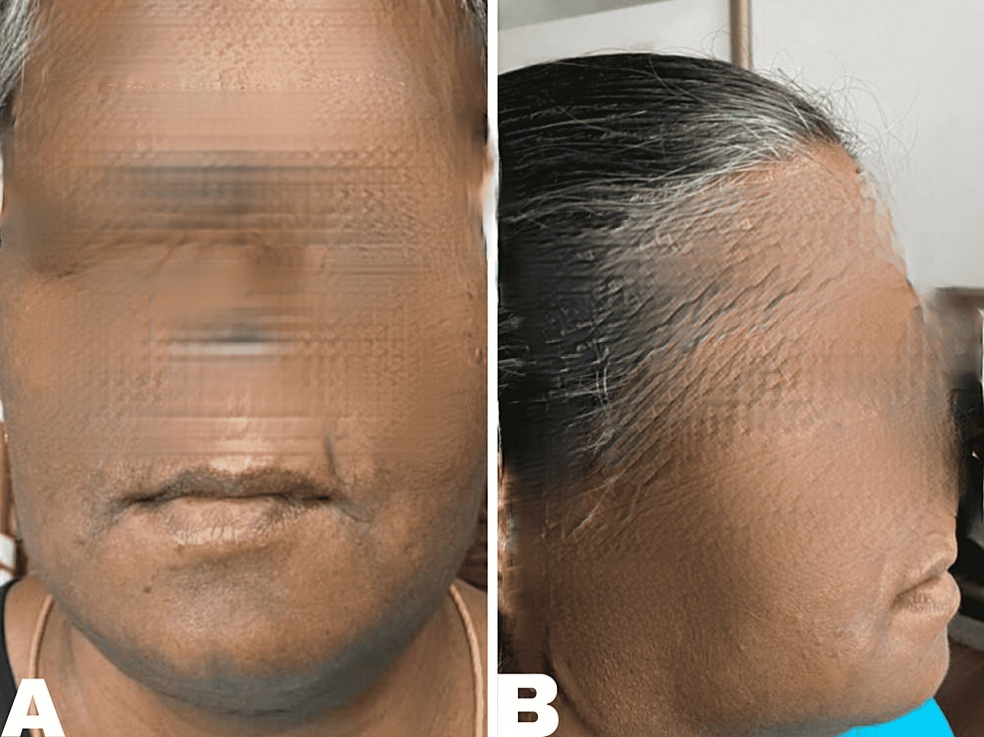
Patient presentation at follow-up after 3 months; A- Front view of the mandible, B- Lateral view of the mandible

Following surgery, the patient was shifted to the intensive care unit where flap monitoring was done for the first 48 hours. The neck wound was inspected on postoperative day 3 and was found to be healthy. The patient was started on speech and swallow therapy. On postoperative day 5, the patient was allowed to mobilize with the help of a walker and was started on oral clear sips on postoperative day 7, which she was able to swallow well. Neck wound staples were removed on postoperative day 12 and the patient was discharged in a stable condition (Figure 4).

**Figure 5:**
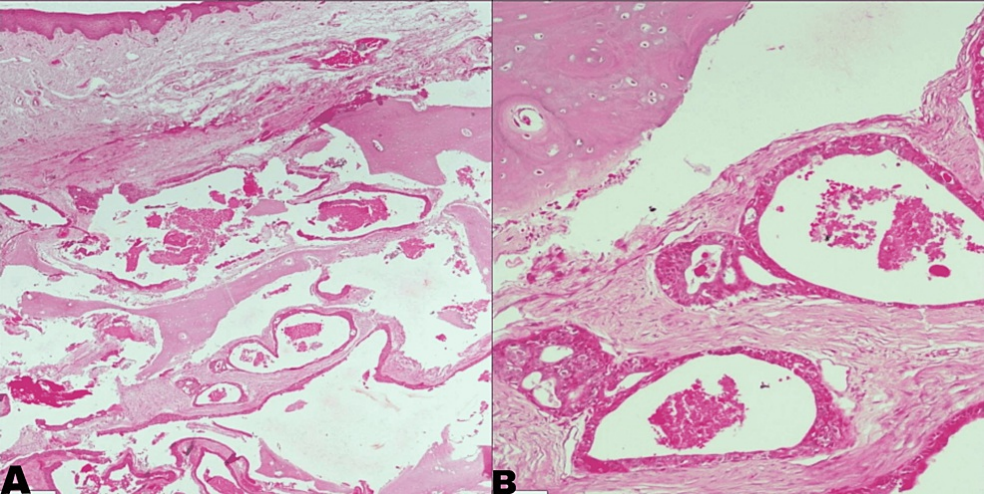
Images (A) and (B) showing sections of mandible with overlying mucosa and an intraosseous cystic neoplasm

**Figure 6:**
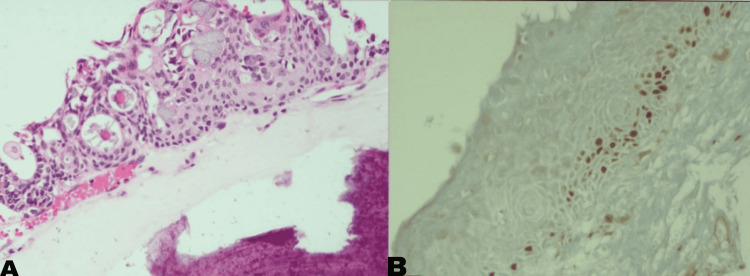
(A) Section showing neoplasm composed of varying mixture of pleomorphic squamoidal cells, intermediate cells, and mucinous cells forming glands; (B) section with Ki-67 immunostaining showing low proliferative index (<5%)

This case was discussed in a multidisciplinary tumor board meeting and, on the arrival of the histopathological report, a final diagnosis of stage 1 (Brookstone and Huvos classification) mucoepidermoid carcinoma of the anterior mandible was made based on the findings (Figures 5-6).

Currently, the patient is four months post-surgery and under regular follow-up in the outpatient department with no evidence of local recurrence or metastasis.

## Discussion

Intraosseous mucoepidermoid carcinoma is a rare neoplasm of the bone, seen more commonly in the mandible and occurs between the third and fifth decades of life, with a female predilection [3]. It is classified as a type IV primary intraosseous carcinoma by Pindborg et al. [5]. These tumors are commonly seen in the posterior aspect of the mandible [4]. However, in the case discussed here, the tumor was found to be in the anterior aspect of the mandible.

Probable theories of the origin of the tumor include the neoplastic transformation of trapped retromolar mucous glands in the mandible; the transformation of remnants of salivary glands lodged in the mandible; the transformation of mucous secreting cells found in the pluripotent epithelium of cysts associated with impacted molars; and the transformation of cells from the maxillary sinus [3]. It commonly presents with symptoms of swelling, pain, destruction of surrounding tissue architecture, and obstruction of the inferior alveolar nerve in chronic cases, leading to altered sensation [4]. However, in the case described, the patient presented with only swelling as the symptom. As it can be easily misdiagnosed as ameloblastoma, keratocystic Odontoceti Christmas tumor, glandular odontogenic cyst, or malignant salivary gland tumor, we must look for some important characteristic features of this tumor, including: (1) the lesion should have an epithelial and a mucinous component; (2) bone margin destruction and cortical bone expansion may be seen; (3) cortical plates are intact; (4) absence of an accompanying lesion in the salivary gland; (5) exclusion of any other primary tumor, the histology of which could resemble the central tumor, and (6) histopathological findings confirming the tumor [6]. Radiologically, a CT scan is the preferred diagnostic modality as it provides information on the extent and size of the tumor [7]. Brookestone and Huvos proposed a three-grade system for the classification of intraosseous mucoepidermoid carcinoma: grade 1- lesions without cortical plate expansion or rupture, grade 2 - lesions with cortical plate expansion but not rupture, and grade 3 - lesions with cortical plate rupture and metastasis to nodes [8]. The features of intraosseous mucoepidermoid carcinoma include having well-defined margins and presenting as either unilocular or multilocular radiolucency [9]. The cortical plate is expanded and encroaches into the surrounding tissues. Findings like internal amorphous sclerotic bone mass and features of malignancy help diagnose the tumor easily [8]. Histologically, mucoepidermoid carcinoma is classified into three subtypes based on: the number of mucous, epidermoid, and intermediate cells; cellular atypia; and cystic formation. Lower-grade tumors show high cystic formation, low atypia, and a high number of mucous cells [10]. High-grade tumors show islands of squamous cells and intermediate cells with high atypia and pleomorphism [10]. A positive mucin stain is also diagnostic for intraosseous mucoepidermoid carcinoma [11]. Fusion of TORC1/MAML2 and MECT1:MAML2 genes in intraosseous mucoepidermoid carcinoma was reported by studies by Li et al. [7] and Brookstone and Huvos [8] and thus they can be used as tumor markers. Intraosseous mucoepidermoid carcinoma can metastasize to the surrounding lymph nodes but rarely to the lung, skin, breast, and cervix [8,9]. Sometimes, metastasis can occur in the ipsilateral clavicle [12]. Treatment modalities include excision, resection, and radiotherapy, but excision is the most preferred treatment modality (17%) [13]. In the case discussed here, segmental mandibulectomy was the treatment modality of choice, thus corresponding with pre-existing data as the preferred treatment modality to ensure the best prognosis for the patient. The prognosis for this neoplasm depends on the histological grade, with the first grade showing the best prognosis and the third grade showing the worst prognosis [4]. In higher-grade tumors, adjuvant chemotherapy and radiotherapy are recommended [8]. Recurrence was seen in only 3% of cases [13].

## Conclusions

Due to the rare occurrence of intraosseous mucoepidermoid carcinoma, more research needs to be done to ensure an increase in the global knowledge of this neoplasm. This will result in an easier and more accurate diagnosis and provide a feasible treatment modality for the patient with the chance of a better prognosis and a lesser chance of recurrence. A multidisciplinary approach must be taken in such cases, involving the pathology department for early detection and accurate diagnosis and a joint approach by the surgical oncology and plastic surgery departments to ensure better prognosis, reconstruction, survival, and quality of life post-treatment.
